# People Like Logical Truth: Testing the Intuitive Detection of Logical Value in Basic Propositions

**DOI:** 10.1371/journal.pone.0169166

**Published:** 2016-12-30

**Authors:** Hiroko Nakamura, Jun Kawaguchi

**Affiliations:** 1 Faculty of Human Informatics, Aichi Shukutoku University, Nagakute, Aichi, Japan; 2 Department of Psychology, Graduate School of Environmental Studies, Nagoya University, Nagoya, Aichi, Japan; Center for BrainHealth, University of Texas at Dallas, UNITED STATES

## Abstract

Recent studies on logical reasoning have suggested that people are intuitively aware of the logical validity of syllogisms or that they intuitively detect conflict between heuristic responses and logical norms via slight changes in their feelings. According to logical intuition studies, logically valid or heuristic logic no-conflict reasoning is fluently processed and induces positive feelings without conscious awareness. One criticism states that such effects of logicality disappear when confounding factors such as the content of syllogisms are controlled. The present study used abstract propositions and tested whether people intuitively detect logical value. Experiment 1 presented four logical propositions (conjunctive, biconditional, conditional, and material implications) regarding a target case and asked the participants to rate the extent to which they liked the statement. Experiment 2 tested the effects of matching bias, as well as intuitive logic, on the reasoners’ feelings by manipulating whether the antecedent or consequent (or both) of the conditional was affirmed or negated. The results showed that both logicality and matching bias affected the reasoners’ feelings, and people preferred logically true targets over logically false ones for all forms of propositions. These results suggest that people intuitively detect what is true from what is false during abstract reasoning. Additionally, a Bayesian mixed model meta-analysis of conditionals indicated that people’s intuitive interpretation of the conditional “*if p then q”* fits better with the conditional probability, *q given p*.

## Introduction

The dual process theory of reasoning [1–5] describes two distinct modes of thinking: one is fast, heuristic, and intuitive, while the other is slow, analytic, and deliberative. According to this theory, when people are faced with a heuristic-logic conflict problem, such as belief-logic conflicts in syllogisms [6], a heuristic-based response comes quickly, and then the reasoners engage in a cognitive effort to inhibit the initial heuristic response and try to endorse a response based on abstract logical rules. It is important to engage in the analytic processes to make logically correct answers in heuristic-logic conflict problems. For example, cognitive capacity, thinking time, instruction to think logically, and thinking style were positively related to identifying logically correct answers in heuristic-logic conflict problems [7–10]. However, it is unclear how people detect conflicts between their initial heuristic answer and logical principles.

Recent reasoning studies suggest that people have implicit knowledge about basic logic or probabilistic principles, which is called *intuitive logic*, and that people can intuitively detect when a heuristic response is in conflict with their intuitive logic [11,12]. Intuitive logic is considered irrelevant with regard to one’s cognitive capacity or deliberate reasoning performance. For example, De Neys and Glumicic [13] showed that even though participants made non-normative responses in heuristic-analytic conflict problems, they needed more time to solve conflict problems than no-conflict problems and that participants’ skin conductance responses increased more in heuristic-logic conflict problems than those in non-conflict problems did [14]. These results suggest that people often fail to explicitly detect normative violations but that they also experience a “gut feeling” that their heuristic response is not fully warranted. Thompson et al. [12,15,16] argued that the analytic process is triggered by the affective response that accompanies intuitive processes. When reasoners are producing answers using intuitive processes, fluent processing gives rise to positive affect, and this positive affect evokes a feeling of rightness (FOR) about their initial answer. The strength of the FOR relates to the extent to which analytic processes are engaged. In other words, a strong FOR reduces the time taken to rethink the answer or the probability that the answer changed. Thompson and Johnson [17] showed that reasoners’ FOR scores were lower for heuristic-analytic conflict problems than for no-conflict problems were and that FOR scores mediated the extent of rethinking their initial answers.

Morsanyi and Handley [18] indicated that people intuitively detect the logical validity of syllogistic reasoning via slight changes in their affective state. Their theory was based on the hedonic marking hypothesis [19], which suggests that perceived fluency triggers positive affect and perceived fluency is triggered by semantic coherence [20]. Morsanyi and Handley [18] argued that the conclusion of a valid syllogism is coherent with the premises, and thus, valid syllogisms are more fluently processed, which leads to a positive affective state. In their experiments, the validity (valid and invalid) and believability (believable, unbelievable, and abstract) of the conclusions of the syllogisms were manipulated. Participants read the premise and conclusion of each syllogism and indicated how much they liked the conclusion by clicking on a smiley/sad face. The results demonstrated that participants liked valid conclusions more than they liked invalid conclusions, as well as preferred believable conclusions more than they preferred unbelievable ones, and they argued that people intuitively perceive the logicality of arguments. However, Klauer and Singmann [21,22] criticized Morsanyi and Handley’s study [18] for their confounding non-logical factors (e.g., some conclusions contained more preferable content and were presented only in valid syllogisms) and they showed that the effect of logical validity on the liking rating disappeared when mean liking for the contents of the conclusions was controlled for. Therefore, it is unclear whether people are intuitively aware of and experience positive feelings about logicality.

The present study aimed to test whether people intuitively detect logical value and have good feelings about logically-true cases. In syllogistic reasoning, a conclusion of a logically-valid argument is always true when its premises are true. If people could intuitively detect logical validity, it is also possible that they intuitively detect whether the conclusion is true or false. To avoid the possible effect of content, the present study used abstract propositions, such as “*if the card has 9 on the left side*, *then it has H on the right side*,” and applied Morsanyi and Handley’s experimental paradigm [18] to test the relationship between logical value and reasoners’ feelings. In the present experiments, participants were sequentially presented with a premise (e.g., “*a card has 9 on the left side and H on the right side*”) and a target (e.g., *9 H*), after which they indicated their feelings. If people intuitively detect logical value, a true case is consistent with the premise and is more fluently processed; therefore, a true case may trigger positive feelings. Conversely, a false case is inconsistent with the premise and is processed more disfluently; therefore, a false case may trigger more negative feelings.

The present study also tested people’s intuitive interpretation of the logical value of a conditional statement, *if p then q*; where *p* is an antecedent and *q* is a consequent. According to mental model theory [23], the logical value of an indicative conditional is equal to the material implication, *¬p or q*, and the conditional is false only in a true antecedent and a false consequent (*TF*) case. While suppositional accounts of conditionals have suggested that people interpret them as the conditional probability of *q given p*. Suppositional accounts have indicated that there is a “defective” truth table for conditionals, also termed the *de Finetti* table, where the conditional is true in a true antecedent and a true consequent (*TT*) case, false in *TF* case and void (neither true nor false) in a false antecedent and a true consequent (*FT*) and a false antecedent and a false consequent (*FF*) case [24,25].

Developmental studies have indicated that response patterns for the conditional truth table develop with age: young children tend to judge the truth value of a conditional to be equal to the conjunctives, *p & q* or the biconditional, *if p then q and if q then p*, and this response declines in older participants and is replaced by the predominant conditional probability response [26]. It was argued that conjunctive responses to conditionals reflects shallow processing and lower cognitive ability, while conditional probability or *de Finetti* Table responses relate to higher cognitive ability [27, 28]. Based on these accounts, three possible hypotheses about intuitive interpretation and liking ratings for conditionals can be proposed:

1. If mental models underlie people’s intuitive logicality, liking ratings for a conditional will show a similar pattern with material implications, *¬p or q*. In this case, participants will like true cases, *TT*, *FT*, and *FF* more than they like the false case, *TF*.
2. If intuitive understanding of a conditional is based on the conditional probability *q given p*, people will like the true case *TT* the best, dislike false *TF*, and the ratings for the void *FT* and *FF* cases will be between those for the *TT* and *TF* cases.
3. If intuitive logical processing is compatible with shallow processing and low cognitive effort, intuitive judgment about conditionals will be similar to that about the conjunctive, and participants will like the *TT* case more than the false *TF*, *FT*, and *FF* cases.

## Experiment 1

Experiment 1 examined whether people intuitively detect logical value by using Morsanyi and Handley’s experimental paradigm [18]. The current experiment presented four logical propositions (conjunctive, biconditional, conditional, and material implication) along with four cases about each proposition, which pertained to whether the antecedent, *p*, and consequent, *q*, both were affirmed or negated: true antecedent and true consequent (*TT*), true antecedent and false consequent (*TF*), false antecedent and true consequent (*FT*), and false antecedent and false consequent (*FF*) cases (Table 1). If participants intuitively detect logical value, true cases will be processed relatively fluently and will lead to positive affective states, while false cases will be processed relatively non-fluently and will lead to negative affective states. Affective states for void (neither true nor false) cases will fall between true and false cases. Experiment 1 also tested whether the intuitive interpretation of conditionals is equal to the material implication, conditional probability, biconditional, or conjunctive forms.

**Table 1:** Truth Table for the Four Logical Forms (Conjunctive, Biconditional, Conditional, Material Implication) and Examples of Materials.

		Target			
Logical form	Proposition	*TT* (*p q*)	*TF* (*p ¬q*)	*FT* (*¬p q*)	*FF* (*¬p ¬q*)
Conjunctive	*p and q*	True	False	False	False
	L and 4	L 4	L 7	K 4	K 7
Biconditional	*If p then q and if q then p*	True	False	False	Void
	If L then 4 and if 4 then L	L 4	L 7	K 4	K 7
Conditional	*If p then q*	True	False	Void	Void
	If L then 4	L 4	L 7	K 4	K 7
(defective truth table)					
Material Implication	*Not-p or q*	True	False	True	True
	Not-L or 4	L 4	L 7	K 4	K 7

Note: Truth value of the biconditional and conditional were based on the suppositional account.

### Materials and Methods

#### Experimental design

This study employed a 4 (logical forms: conjunctive, biconditional, conditional, and material implication) by 4 (targets: *TT*, *TF*, *FT*, and *FF*) within-participants design.

#### Participants and ethics statement

Twenty-nine Japanese undergraduate students (11 females and 18 males, *M*age = 18.4, *SD*age = 0.68) participated in exchange for partial course credit. Participants were novices at using formal logic and they attended the experiment individually.

Verbal informed consent was obtained from each participant prior to the study initiation, and all data were anonymized in order to maintain the confidentiality of our subjects. As all research data were anonymous and the study was deemed to involve only minimal risk, no written informed consent procedure was used in the study. This study and the consent procedure were approved by the ethics committee of the Department of Psychology, Nagoya University Graduate School of Environmental Studies.

#### Materials

We created six hypothetical cards with one of two letters on the left side and one of two numbers on the right side (e.g., *the card has L or K on the left side and it has 4 or 7 on the other side*). Each of the six cards used a different letter and number combination, and we designed experimental blocks based on each card. For all six cards, we created four logical forms (conjunctive, biconditional, conditional, and material implication) that described a statement about the card (e.g., *if the card has L on the left side*, *then it has 4 on the right side*). There were four targets for each proposition, which pertained to whether the antecedent and consequent (or both) were affirmed or negated (e.g., *L 7*, *K 4*). For each of the six cards, the four logical forms were presented with each of the four targets. This resulted in 16 trials for each block of six cards, and 96 trials in total. Both the liking rating and truth table tasks used the same card blocks but in a different order. For each participant, the six card blocks were presented in a random order for each task, and the 16 trials in each block were also presented in random order.

#### Procedure

Stimulus presentation and data collection were carried out on a computer using the PsyScope X program (PsyScope X Build 77, http://psy.ck.sissa.it/) and a response button box. Participants were seated in front of a PC screen and were asked to respond to each task by pressing the button on the button box. The experimental procedure was based on the procedure used by Morsanyi and Handley [18]. The experiment consisted of two parts: the first part was the liking rating task, and the second was the truth table task. In the first part, participants were presented with a proposition and a target, and relying on their intuition, they were asked to indicate how they felt using a 5-point Likert-type scale ranging from 1 (“don’t like it”) to 5 (“like it”). The scale was presented with line drawings of smiley/sad faces. In the second part of the experiment, participants were presented with the same proposition and target again, and they had to decide whether the proposition on the card was *true*, *false*, or *neither true nor false*.

**Fig 1:**
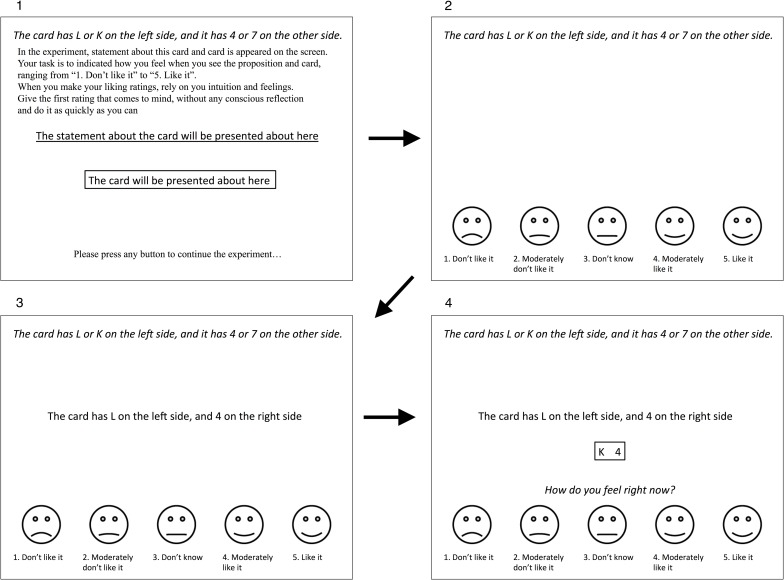
Example of the Liking Rating Task in Experiment 1 (Conjunctive Form and *FT* Target).

The experimental procedure for the liking rating task comprised two phases (Fig 1). First, in the instruction phase, the statement about the letter and number on the card was presented, and instructions for the liking rating task appeared at the center of the screen. Participants were asked to confirm the experimental procedure and the letter and number on the card, and press any key to continue the task. Second, in the liking rating phase, participants indicated how they felt when they were presented with the proposition and card. During the rating phase, a statement about the letter and number on the card was continuously presented at the top of the screen. A 5-point Likert-type scale with line drawings of smiley/sad faces was also continuously presented at the bottom of the screen during the rating phase. In the liking rating phase, at first, a proposition was presented in the center of the screen. After 2000 ms, the target was presented, together with the question “How do you feel right now?” The proposition and target remained on the screen until participants responded, after which, the proposition and card were cleared. The next proposition was presented 2000 ms later. The rating phase continued until 16 trials for each card block were conducted; then the next card block was presented. After participants finished the liking rating task, they had a short break and then started the truth table task.

The procedure for the truth table task was almost the same as that for the liking rating task: The proposition and the card were sequentially presented on the screen, and participants were asked to decide whether the sentence about the card was logically true, false, or neither true nor false. In the truth table task, participants were instructed to think carefully and logically and to take as much time as they needed.

### Results

#### Liking rating task

**Fig 2:**
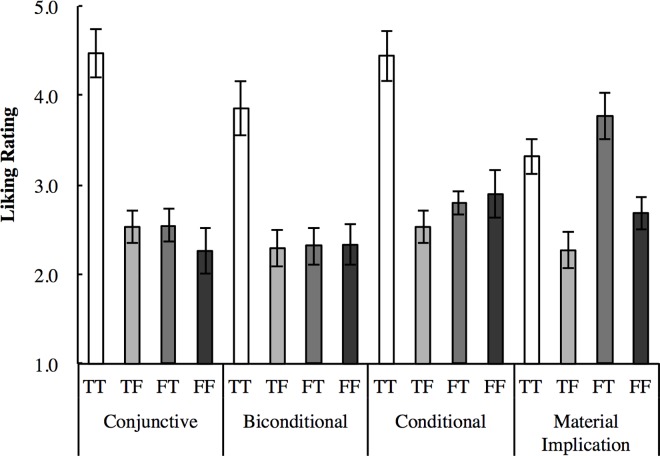
Mean Liking Ratings and Cousineau-Morey Difference Adjusted 95% Confidence Intervals for the Four Logical Forms and Targets in Experiment 1.

Fig 2 shows the mean liking rating for each logical form and case. An analysis of variance (ANOVA) was conducted with the logical forms and targets as within-participants variables. There were main effects for logical form: *F*(3, 84) = 5.56, *p* = .002, *η*_*G*_^*2*^ = 0.37; target: *F*(3, 84) = 38.42, *p* < .001, *η*_*G*_^*2*^ = 0.35; and an interaction between logical form and target: *F*(9, 252) = 17.57, *p* < .001, *η*_*G*_^*2*^ = 0.15.

The simple main effect of the target was significant for each form type: conjunctive: *F*(3, 84) = 42.00, *p* < .001, *η*_*G*_^*2*^ = 0.51; biconditional: *F*(3, 84) = 28.25, *p* < .001, *η*_*G*_^*2*^ = 0.32; conditional: *F*(3, 84) = 30.00, *p* < .001, *η*_*G*_^*2*^ = 0.47; and material implication: *F*(3, 84) = 18.91, *p* < .001, *η*_*G*_^*2*^ = 0.35. Multiple comparisons were conducted with Shaffer’s modified sequentially rejective Bonferroni procedure. For the conjunctive form, the *TT* was liked more than the *TF*, *FT*, and *FF*. For the biconditional form, the *TT* was rated higher than the *TF*, *FT*, and *FF*. For the conditional form, the *TT* was rated higher than the *TF*, *FT*, and *FF*; while the *FT* was rated higher than the *TF*. For the material implication form, the *FT* was rated higher than the *TT*, *TF*, and *FF*; the *TT* was rated higher than the *TF* and *FF*; and the *FF* was rated higher than the *FT*.

The simple main effect of logical form was significant for the *TT*: *F*(3, 84) = 16.05, *p* < .001, *η*_*G*_^*2*^ = 0.22; *FT*: *F*(3, 84) = 19.23, *p* < .001, *η*_*G*_^*2*^ = 0.32; and *FF*: *F*(3, 84) = 5.80, *p* = .001, *η*_*G*_^*2*^ = 0.08; but not for *TF*: *F*(3, 84) = 2.15, *p* = .101, *η*_*G*_^*2*^ = 0.02. For the *TT*, the biconditional, conditional and conjunctive forms were rated higher than the material implication form, and the conditional and conjunctive forms were rated higher than the biconditional form. For the *FT*, the material implication form was rated higher than the biconditional, conditional, and conjunctive forms, and the conditional form was rated higher than the biconditional and conjunctive forms. For the *FF*, the conditional and material implication forms were rated higher than the biconditional and conjunctive forms.

#### Truth table task

Table 2 shows the percentages for the response categories in the truth table task. To test the differences between response categories for each target in each logical form, a chi-square and residual analyses were conducted. For the conjunctive form, the *TT* case was more frequently judged as true, while the *TF*, *FT* and *FF* cases were more frequently judged as false: *χ*^*2*^(6) = 671.1, *p* < .001, *Cramer’s V* = .694. For the biconditional form, the *TT* case was more frequently judged as true, the *TF* and *FT* were more frequently judged as false, and the *FF* was more frequently judged as void: *χ*^*2*^(6) = 869.7, *p* < .001, *V* = .794. For the conditional form, the *TT* case was more frequently judged as true, the *TF* case was more frequently judged as false, and the *FT* and *FF* cases were more frequently judged as void: *χ*^*2*^(6) = 900.9, *p* < .001, *V* = .807. For the material implication form, the *TT*, *FT*, and *FF* cases were more frequently judged as true, and the *TF* case was more frequently judged as false: *χ*^*2*^(6) = 413.1, *p* < .001, *V* = .545.

**Table 2:** Percentages for the Response Categories (True, False, and Void) in the Truth Table Task in Experiment 1.

		Response		
Logical form	Target	True	False	Void
Conjunctive	*TT*	98.3	1.1	0.6
	*TF*	1.1	97.1	1.7
	*FT*	2.3	96.0	1.7
	*FF*	0.6	88.4	11.0
Biconditional	*TT*	97.1	2.9	0.0
	*TF*	0.6	93.1	6.4
	*FT*	0.6	86.8	12.6
	*FF*	5.2	28.3	66.5
Conditional	*TT*	97.7	0.6	1.7
	*TF*	0.0	95.4	4.6
	*FT*	4.1	23.4	72.5
	*FF*	6.9	20.1	73.0
Material implication	*TT*	78.6	16.2	5.2
	*TF*	2.9	96.0	1.2
	*FT*	96.0	2.3	1.7
	*FF*	71.7	23.1	5.2

## Discussion

Experiment 1 tested whether participants intuitively detected the logical value of abstract propositions and tested the reasoners’ intuitive interpretation of conditionals. We predicted that true cases were fluently processed and evoked positive feelings more than disfluent false cases. As we expected, participants liked the true cases more than they did the false cases in all four logical forms, and the results suggested that participants had intuitively distinct, and logically consistent and inconsistent cases.

We also observed differences in the pattern of liking ratings between the four logical forms. According to the suppositional account of conditionals [24,27,29], the conditional is void, “i.e., neither true nor false”, in false antecedent cases. We predicted that if people’s understanding of a conditional is equal to the conditional probability, the liking rating of the conditional is different from that of conjunctive or material implication cases and the liking rating for the void (*FT*, *FF*) cases would be in between those for true and false cases. In the explicit truth table task, the results corresponded better with the suppositional account of conditionals: Participants judged false antecedent cases as void in the conditional case, and the *FF* case in the biconditional form was also judged as void. In the intuitive liking rating task, participants liked true *TT* cases more than void cases, both in the conditional and biconditional forms, and the *FT* case was rated higher than the false *TF* case in the conditional form. However, there were no differences between the *FF* case and the false cases, both in the conditional and biconditional forms.

One possible explanation for these results is that a matching bias might have affected the liking rating. A matching bias is the tendency to evaluate cases as relevant to the conditional when the lexical content of a case matches what is explicitly mentioned in the conditional [30,31]. For example, in the conditional statement: “*if the card has* *L* *on the left side*, *then it has* *4* *on the right side*,*”* “*L”* and *“4”* are matching cases and judged as relevant to the conditional, while “*K”* and *“7”* are mismatching cases and judged as irrelevant to the conditional. Whereas, in the statement, “*if the card has not-**K* *on the left side*, *then it has not-**7* *on the right side”* “*K”* and *“7”* are matching cases and are judged as relevant to the conditional, while “*L”* and *“4”* are mismatching cases and the tendency is to judge them as irrelevant to the conditional. In the present study, the true, or the *TT* case was a double matching case in all four logical propositions. Therefore, it was possible that participants might like matching cases more as they were perceived as relevant and consistent with the proposition; whereas they might dislike mismatching cases since they were perceived as irrelevant and inconsistent with the proposition.

In summary, the results of Experiment 1 implied that participants intuitively judged the logical value of propositions, and that logically true cases were more fluently processed and induce positive feelings more than false or void cases did. However, it is possible that matching bias affected the liking judgment. In Experiment 2, we tested whether people like matching or logically true cases.

## Experiment 2

Experiment 2 tested the effect of matching bias and logicality on the intuitive process of reasoning. For this purpose, Experiment 2 applied a negated paradigm of the conditional [31], which presented four forms of a conditional with the presence or absence of a negative component (Table 3). Evans [32] showed that *irrelevant* responses were highest for the double-mismatch *¬p ¬q* case, lowest for the double-match *p q* case, and that irrelevant responses to the single-match *p ¬q* and *¬p q* cases were in-between the two. If matching affects the intuitive process of reasoning, a matching case will be perceived as relevant to the conditional and may be more fluently processed. Therefore, a double matching case may induce more positive feelings than mismatching cases, and single matching cases may be rated more positively than double mismatching cases. On the other hand, if logicality affects the liking rating, liking ratings would be higher for the true *TT* cases than for the false *TF* cases, and the void *FT* and *FF* cases would be rated at an intermediate level, regardless of matching.

**Table 3:** A Negated Paradigm of a Conditional With an Example of the Conditional Statements and Targets.

	Target			
Conditional	TT	TF	FT	FF
*If p then q*	*p q*	*p ¬q*	*¬p q*	*¬p ¬q*
If L then 4	L 4 ++	L 7 +-	K 4 -+	K 7 --
*If p then ¬q*	*p ¬q*	*p q*	*¬p ¬q*	*¬p q*
If L then not-4	L 7 +-	L 4 ++	K 7 --	K 4 -+
*If ¬p then q*	*¬p q*	*¬p ¬q*	*p q*	*p ¬q*
If not-L then 4	K 4 -+	K 7 --	L 4 ++	L 7 +-
*If ¬p then ¬q*	*¬p ¬q*	*¬p q*	*p ¬q*	*p q*
If not-L then not-4	K 7 --	K 4 -+	L 7 +-	L 4 ++

Note

++ double matching

+- antecedent matching

-+ consequent matching

-- double mismatching.

In addition, we tested the relationship between the deliberate reasoning and intuitive reasoning processes. It has been suggested that the suppositional interpretation of conditionals requires cognitive effort and cognitive capacity [25,27,28]. Furthermore, Barrouillet et al. [26] showed that the *de Finetti* table (defective truth table) responses for conditionals, which correspond with suppositional interpretation, were acquired at a later stage in development. Therefore, we counted the number of *de Finetti* table responses in the truth table task as an index of participants’ explicit reasoning performance. Moreover, we administered the Rational-Experiential Inventory (REI) [33,34], which measures participant’s rationality (“i.e., the extent to which a person enjoys and engages in problem solving”) and experientiality (“i.e., the extent to which a person relies on intuition and past experience”) thinking styles. Rationality is related to working memory capacity or the ability to engage in deliberate thinking and perform cognitive tasks [10]. However, previous studies indicated that intuitive logic is irrelevant to one’s cognitive capacity or explicit reasoning performance [13,14,18]. Therefore, if the liking rating task reflects the intuitive process of reasoning, there would be no positive correlation between the liking rating performance and truth-table task performance or the rationality score.

### Materials and Methods

#### Experimental design

Experiment 2 employed a 4 (conditionals: *if p then q*, *if p then ¬q*, *if ¬p then q*, and *if ¬p then ¬q*) by 4 (targets: *TT*, *TF*, *FT*, and *FF*) within-participants design.

#### Participants and ethics statement

Twenty-seven Japanese undergraduate students (13 males and 14 females, *M*age = 19.0, *SD*age = 1.37) participated in the study in exchange for partial course credit. Participants were novices in formal logic and attended the experiment individually.

Verbal informed consent was obtained from each patient prior to study participation, and all data were anonymized. As all research data were anonymous and the study was deemed to involve only minimal risk, no written informed consent procedure was introduced. The study and consent procedure were approved by the ethics committee of the Department of Psychology, Nagoya University Graduate School of Environmental Studies.

#### Materials

As in Experiment 1, we created hypothetical cards with letters and numbers. In Experiment 2, we created 12 hypothetical cards. Each of the 12 cards used different letter and number combinations, and we created experimental blocks based on each card. The card described the conditionals and differed in terms of whether the antecedent and consequent (or both) were affirmed or negated, for example, “*if the card has not-L on the left side*, *then it has 4 on the right side*.” We created four targets for each conditional in terms of whether the antecedent and consequent (or both) were affirmed or negated (*TT*, *TF*, *FT*, and *FF*).

For the 12 cards, each of the four conditionals was presented with each of the four targets. This resulted in 16 trials for each of the 12 card blocks and 192 trials in total. In Experiment 2, to eliminate the possible effect of repetition, we applied different card blocks for the liking rating and truth table tasks, where four card blocks were randomly assigned to the liking rating task and eight card blocks were randomly assigned to the truth table task. Therefore, participants responded to four card blocks with 64 trials in the liking rating task, and eight card blocks with 98 trials in the truth-table task. For each participant, the presentation order of the card blocks was counter-balanced and the 16 trials for each card block were randomized.

We used the Japanese version of the REI, which is a 38-item self-report questionnaire that measures rationality and experientiality thinking styles [33,34].

#### Procedure

As in Experiment 1, the experimental procedure was based on Morsanyi and Handley’s studies [18]. The experiment consisted of two parts: The first part was the liking rating task, and the second part was the truth table task. In the liking rating task, 64 conditionals and targets were presented and participants were asked to indicate how they felt, using a 5-point Likert-type scale with line drawings of smiley/sad faces (e.g., 1 “don’t like it” to 5 “like it”). In the truth table task, 128 conditionals and targets were presented and participants judged whether the sentence about the target card was “true,” “false,” or “neither true nor false.” The experimental procedure for the liking rating and truth table tasks was the same as those used in Experiment 1. Lastly, participants were asked to complete the REI.

### Results

#### Liking rating task

**Fig 3:**
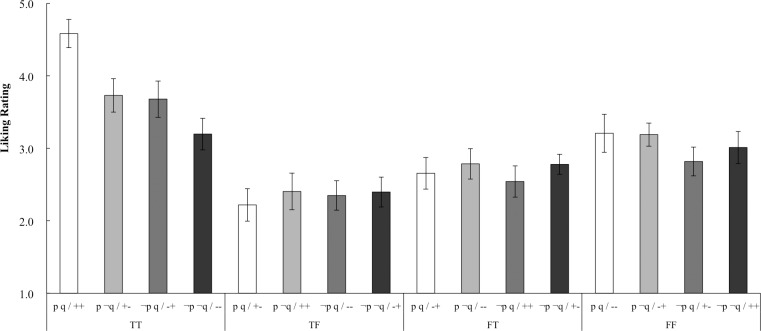
Mean Liking Ratings and Cousineau-Morey Difference Adjusted 95% Confidence Intervals for the Four Targets and Matching in Experiment 2. Note: ++ double matching, +- antecedent matching, -+ consequent matching,—double mismatching

Fig 3 shows the mean liking rating for each target and matching. To test the possible effects of truth-value and matching, an ANOVA was conducted with the targets (*TT*, *TF*, *FT*, and *FF*) and matching (double match *p q*, antecedent match *p* ¬*q*, consequent match ¬*p q*, and double mismatch ¬*p ¬q*) as within-participant variables. Significant effects were found for the targets: *F*(3, 78) = 99.70, *p* < .001, *η*_*G*_^*2*^ = 0.18; matching: *F*(3, 78) = 7.34, *p* < .001, *η*_*G*_^*2*^ = 0.01; and the interaction between targets and matching: *F*(9, 234) = 10.00, *p* < .001, *η*_*G*_^*2*^ = 0.04. The simple main effect of the targets was significant for each matching: double match: *F*(3, 78) = 89.17, *p* < .001, *η*_*G*_^*2*^ = 0.38; antecedent match: *F*(3, 78) = 25.72, *p* < .001, *η*_*G*_^*2*^ = 0.16; consequent match: *F*(3, 78) = 31.46, *p* < .001, *η*_*G*_^*2*^ = 0.17; and double mismatch: *F*(3, 78) = 10.65, *p* < .001, *η*_*G*_^*2*^ = 0.06. Multiple comparisons with Shaffer’s modified sequentially rejective Bonferroni procedure were conducted. For the double matching *p q*, the *TT* was rated higher than the *TF*, *FT*, and *FF*; the *FF* was rated higher than the *TF* and *FT*; and the *FT* was rated higher than the *TF*. For the antecedent matching, *p ¬q*, the *TT* was rated higher than the *TF*, *FT*, and *FF*; the *FF* was rated higher than the *TF* and *FT*; and the *FT* was rated higher than the *TF*. For the consequent matching, *¬p q*, the *TT* was rated higher than the *TF*, *FT*, and *FF*; while the *FF* was rated higher than the *TF*. For the double mismatching, *¬p ¬q*, the *TT*, *FT*, and *FF* were rated higher than the *TF*. The simple main effect of the matching was significant for the *TT*: *F*(3, 78) = 34.27, *p* < .001, *η*_*G*_^*2*^ = 0.18, and multiple comparisons showed that double matching, *p q*, was rated higher than *p ¬q*, *¬p q*, and *¬p ¬q*; while *p ¬q* and *¬p q* were rated higher than the double mismatching *¬p ¬q*. Finally, the simple main effect of the matching was not significant for the *TF*: *F*(3, 78) = 0.73, *p* = .535, *η*_*G*_^*2*^ = 0.005; *FT*: *F*(3, 78) = 1.24, *p* = .302, *η*_*G*_^*2*^ = 0.008; and *FF*: *F*(3, 78) = 2.65, *p* = .054, *η*_*G*_^*2*^ = 0.017.

#### Truth table task

Table 4 shows percentages for the response categories in the truth table task. To test the differences between the response categories for each target in each conditional statement, chi-square and residual analyses were conducted. In all four conditional statements, the *TT* was more frequently judged as true, the *TF* was more frequently judged as false, and the *FT* and *FF* were more frequently judged as void: *if p then q*: *χ*^*2*^(6) = 1171.7, *p* < .001, *V* = .823; *if p then ¬q*:*χ*^*2*^(6) = 1139.7, *p* < .001, *V* = .814; *if ¬p then q*:*χ*^*2*^(6) = 890.4, *p* < .001, *V* = .718; *if ¬p then ¬q*: *χ*^*2*^(6) = 651.7, *p* < .001, *V* = .614.

**Table 4:** Percentages for the Response Categories (True, False, and Void) in the Truth Table Task in Experiment 2.

			Response		
Conditionals	Target	Matching	True	False	Void
*If p then q*	TT	p q/++	99.1	0.9	0.0
	TF	p ¬q/+-	0.0	92.6	7.4
	FT	¬p q/-+	2.8	22.2	75.0
	FF	¬p ¬q/--	9.3	11.1	79.2
*If p then* ¬*q*	TT	p ¬q/+-	93.1	2.3	4.6
	TF	p q/++	3.2	92.6	4.2
	FT	¬p ¬q/--	6.9	8.3	84.3
	FF	¬p q/-+	14.4	6.9	77.3
*If* ¬*p then q*	TT	¬p q/-+	92.1	1.4	6.0
	TF	¬p ¬q/--	4.2	81.0	14.8
	FT	p q/++	6.0	34.7	59.3
	FF	p ¬q/+-	9.3	10.2	80.1
*If* ¬*p then* ¬*q*	TT	¬p ¬q/--	77.3	5.1	17.6
	TF	¬p q/-+	6.9	82.9	10.2
	FT	p ¬q/+-	12.0	16.7	71.3
	FF	p q/++	32.4	10.2	57.4

Note

++ double matching

+- antecedent matching

-+ consequent matching

-- double mismatching

To test the possible effect of matching, for each matching in each target, chi-square and residual analyses were conducted. For the *TT* target, true response was more frequently observed in the double matching, *p q*, than in the double mismatching, ¬*p* ¬*q*, *χ*^*2*^(6) = 32.8, *p* < .01, *V* = .196. For the *TF* target, false response was more frequently observed in the antecedent matching, *p ¬q*, than in the double mismatching, *¬p ¬q*, *χ*^*2*^(6) = 15.6, *p* < .05, *V* = .133. For the *FT* target, void response was more frequently observed in the double mismatching, *¬p ¬q*, than in the double matching, *p q*, *χ*^*2*^(6) = 31.3, *p* < .05, *V* = .190. For the *FF* target, the void response was less frequently observed in the double matching, *p q*.

#### Relationship between the liking rating and truth table responses, and REI

To test whether participants’ thinking style and explicit reasoning performance were related to the intuitive liking ratings, correlations between response patterns for the truth table, REI score, and liking rating tasks were analyzed (Table 5). In the present study, participants completed the truth table task for 32 conditionals, and the average number of *de Finetti* Table response patterns was 16.31 (*SD* = 9.84). A correlation analysis between the number of *de Finetti* Table responses and the liking ratings indicated that the liking ratings for the *TT* in the antecedent matching *p ¬q*, consequent matching ¬*p q*, and double mismatching ¬*p ¬q* were positively correlated with the number of *de Finetti* Table responses in the truth table task. As for the REI score, the rationality score was positively correlated with the liking rating for the *TT* in the antecedent matching *p ¬q*, while there were no statistically significant correlations between the experientiality score and the liking rating.

**Table 5:** Correlations Between the Number of *De Finetti* Table Responses in the Truth Table Task, Rational-Experiential Inventory Scores, and Liking Ratings in Experiment 2.

				Rational-Experiential Inventory scores		
Targets	Matching	*de Finetti*		Experientiality	Rationality	
TT	p q/++	-.01		.05	.04	
	p ¬q/+-	.40	*	.20	.50	*
	¬p q/-+	.49	*	-.03	-.15	
	¬p ¬q/--	.40	*	.22	-.17	
TF	p ¬q/+-	-.06		-.15	.11	
	p q/++	.11		-.11	.11	
	¬p ¬q/--	.08		-.16	.05	
	¬p q/-+	.08		-.27	-.05	
FT	¬p q/-+	.10		-.25	-.16	
	¬p ¬q/--	.21		-.09	-.01	
	p q/++	-.11		-.22	-.04	
	p ¬q/+-	-.02		-.21	.08	
FF	¬p ¬q/--	.11		.22	-.08	
	¬p q/-+	.34		.03	.10	
	p ¬q/+-	-.01		.22	.34	
	p q/++	-.06		-.12	.24	

* *p* < .05.

Note

++ double matching

+- antecedent matching

-+ consequent matching

-- double mismatching

### Discussion

Experiment 2 tested whether the intuitive process of conditional reasoning was affected by logic or matching bias. The results of the liking rating task indicated that both logic and matching affected participants’ feelings. As for the effects of logicality, liking ratings of true cases were higher than those for false cases in all matching patterns. Therefore, participants might have been intuitively aware of the logical value of conditionals, regardless of matching. Although liking ratings for void cases were lower than those for true cases and higher than those for false cases in all matching patterns, some of these differences did not reach statistical significance. Since void cases are neither true nor false, the interpretation of their truth value might be more difficult than that for true or false cases; therefore, the processing fluency and liking ratings of void cases was unstable.

The effects of matching were observed in true cases, where participants liked the double matching *p q* target more than they did the other three targets, and the liking ratings for the double mismatching *¬p ¬q* target were lower than those for the other three targets. On the other hand, there were no statistically significant effects of matching in false or void cases. According to logical intuition theory (e.g., De Neys [11]), even if a reasoner makes a logically inappropriate response in an explicit reasoning task, people intuitively detect the heuristic-logic conflict. In the present study, the effect of conflict between matching heuristic and logical values was significant only in logically true cases, where people liked the no-conflict double matching target more than the conflict mismatching targets, while the effect of heuristic-logic conflict was not obvious in false or void cases. One possible interpretation is that true cases were consistent with the premise and might be more fluently processed; therefore, participants could easily perceive the surface inconsistency caused by mismatching in true cases. Conversely, false or void cases were inconsistent with the premise, and the effect of surface inconsistency by mismatching might be relatively low in these cases.

The results of the truth table task were compatible with the suppositional theory of conditionals, where the modal response pattern adhered to the *de Finetti* table for all forms of conditionals. The effects of the matching bias were also observed on the truth table task: The void response was more frequently observed for the double mismatch *¬p* ¬*q* target than for the double match *p q* target. These results are compatible with those of previous studies [27, 32, 35] that indicated that irrelevant responses occurred when targets are mismatched with the explicit lexical content.

The correlations between the liking rating and truth table tasks indicated that participants who made *de Finetti* Table responses in the truth table task preferred true cases more, even with mismatching targets, than did participants who made fewer *de Finetti* Table responses. Thompson & Johnson [17] indicated individual differences in metacognitive awareness of logicality and argued that some reasoners were more likely to master logical principles and had more normative intuitions. Thus, participants who made more normative responses in explicit reasoning tasks might be more likely to activate logical intuition and be less affected by non-normative factors, such as matching, in intuitive processes. Note that we did not observe significant correlations between performance of truth table tasks and liking ratings in the false and void cases. As indicated earlier, true cases might be more sensitive for mismatching-induced disfluency. Therefore, we observed correlations between liking ratings and truth table performances only in *TT* cases. Furthermore, the correlation between the REI score and liking rating was relatively small. Since the rationality score is related with cognitive capacity and engaging in deliberate thought [10], it is possible that the effect of cognitive engagement in the liking rating was relatively low.

### A Bayesian mixed meta-analysis on the intuitive processing of conditionals

Singmann et al. [22] replicated Morsanyi and Handley’s study [18] and conducted a Bayesian mixed meta-analysis. They showed that liking ratings of syllogistic reasoning were best predicted by random effects for participants and items but not by a fixed effect of logical forms. To examine the intuitive interpretation of conditionals, the present study analyzed whether the liking ratings for conditional statements are predicted best by matching or logical interpretation (e.g., conditional probability, conjunctive, or material implication forms). Note that we did not include the biconditional in the present analysis.

We conducted a Bayesian mixed meta-analysis on the liking ratings for the conditional statements in Experiments 1 and 2. We calculated the Bayes Factor (BF) by comparing the null model, which contained a random intercept of the participants only, and had a no fixed effect. The models differed in both their fixed effects and random effects structures.

As for the fixed effects, we created the following four models about the interpretation of the conditional: (1) matching, (2) suppositional, (3) conjunctive and (4) material implication.

1. The matching model had three levels: matching (*p q*), mismatching (¬*p* ¬*q*), and partially matching (¬*p q* and *p* ¬*q*).
2. The suppositional model had three levels: true (*TT*), false (*TF*), and void (*FT* and *FF*) targets.
3. The conjunctive model had two levels: true (*TT*), and false (*TF*, *FT*, and *FF*) targets.
4. The material implication model had two levels: true (*TT*, *FT*, and *FF*), and false (*TF*) targets.

In each logical interpretation (e.g., suppositional, conjunctive, and material implication), there were five different fixed effects structures pertaining to their combination with matching. For example, suppositional interpretation had the following five fixed effect structures: (1) none (no fixed effects structure), (2) matching, (3) suppositional, (4) effect of both suppositional and matching (suppositional + matching), and (5) interaction between suppositional and matching (suppositional * matching). Similarly, both conjunctive interpretation and material implication interpretation also had five fixed effect structures.

As for the random effect structures, we employed random effects of participants, items (letter and number pairs) and experiment (Experiment 1 and 2). It is important to note that we could have made numerous models for different random effects structures, but in the present analysis we were interested in whether the liking rating is best predicted by matching or logical interpretation (e.g., suppositional, conjunctive, or material implication interpretations), and which random effects affect these factors. There were individual differences in logical reasoning performance and metacognitive awareness of logicality [17], and it is likely that there were individual differences in liking rating performance. We predicted that models that include individual differences (e.g., random intercept of participants, participants’ random slope for effect of matching, and logical interpretations) were more plausible than models that did not include the random effect of participants. On the other hand, the present experiments used abstract letter-number items, and both experiments employed similar experimental procedures. Therefore, it is likely that the effects of items and experiments on the liking rating were relatively low. Consequently, there might be no differences between models that include the effect of items and experiments (e.g., items random intercept and experiments’ random slope for the effect of logical interpretations), and models that did not include the random effect of items and experiments. To test the effect of participants, items and experiments on liking rating, we created the following four random effect terms: (R1) participants; (R2) participants and items; (R3) participants and experiments; (R4) participants, items, and experiments. We employed both random intercept and random slope structures of each random effect term for each fixed effect. For example, suppositional interpretation models had the following five random slope structures for each random effect term: (1) intercept only, (2) random slopes for the suppositional, (3) random slopes for matching, (4) random slopes for the suppositional + matching and (5) random slopes for the interaction between the suppositional * matching. To sum up, the present analysis included five fixed effect structures, four random effect terms, and five random slopes for each logical interpretation (suppositional, conjunctive, and material implication), and yielded 300 models in total.

The analysis was performed using the *R* statistical software [36] with the *Bayes Factor* package [37]. All models were compared with the denominator model, which had no fixed effect and only the random intercept of participants. The interpretation of the Bayes factor was based on Jeffrey’s standard scale of interpretation [38].

The summary of the results of the Bayesian mixed meta-analysis have been presented in Table 6. F1–F4 are the fixed effects of matching or logical interpretations with random intercept only models. F5–F7 are the largest BF models in matching and each logical interpretation. The full results of the Bayesian mixed model meta-analysis have been reported in the supporting information (S1 File).

**Table 6:** Summary of the Results of the Bayesian Mixed Model Meta-analysis [log(BF)].

			Random Effects			
			R1	R2	R3	R4
	Fixed Effects	Random Slopes	Participants	Participants and Items	Participants and Experiments	Participants, Items, and Experiments
F0	None	Intercept only	0 *(null model)*	-14.6	-1.1	-16.6
F1	Matching	Intercept only	59.7	45.5	58.6	43.5
F2	Suppositional	Intercept only	274.6	261.7	273.8	259.9
F3	Conjunctive	Intercept only	245.1	232	244.2	230.1
F4	MI	Intercept only	59.7	45.5	58.6	43.5
F5	Suppositional * Matching	Suppositional + Matching	**434.4**	389.6	424.5	380.9
F6	Conjunctive * Matching	Conjunctive + Matching	390.2	349.4	382.3	342.4
F7	MI + Matching	MI * Matching	410.1	238.7	197.5	253.3

Note. All models are compared against the denominator (null) model, which included no fixed effect structure and had only the random intercept of the participants.

* interaction

+ main effects and no interaction

Regarding fixed effects, models that included both effects of logicality and matching (F5–F7) showed a larger BF value as compared to the effect of logicality or matching only models (F1–F4). The results indicated that both matching heuristics and logical principles affect participants’ liking rating. The overall best model had the fixed effects of suppositional * matching (F5) and participants random slopes for both effects of the suppositional and matching. In addition, within the main effect only models (F1–F4), the suppositional model (F2) showed the largest BF value. Therefore, models that included three-values (e.g., true, false, and void) fit better with participants’ intuitive interpretation of the conditional as compared to the two-valued logic (e.g., conjunctive or material implication).

As for the random effects and random slopes, the random effect for the participants only models (R1) showed a larger BF as compared to models that included the random effects for items and experiments (R2–R4) in the fixed effect structures that included logical interpretations (F2–F7). Further, the models that included participants random slopes of logical interpretations and matching (F6–F7) showed a larger BF value than random intercept only models (F1–F4). These results indicated that there were individual differences in the effect of logical interpretations and matching on liking rating, while the effects of items and experiments on the liking rating were relatively low. Note that, in the fixed effect structure of matching (F1), models that included the random effect for experiments (R3–R4) showed larger BF values than the random effect of participants only model (R1). These results reflected that matching was manipulated within Experiment 2.

**Fig 4:**
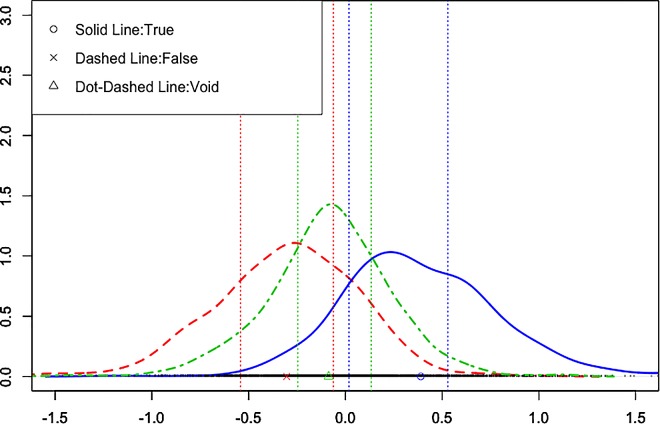
Density and Credible Intervals of 1000 Posterior Samples for the relevant fixed effects of the Maximal Random Effect Structure for the Fixed Effects of the Suppositional * Matching Model. Vertical lines depict the 25–75% posterior probability intervals.

To further examine the effect of logicality, we obtained 1000 samples for each parameter from the posterior distribution of suppositional * matching in maximal random effect structure model: fixed effects of suppositional and matching interaction with participants, and items’ and experiments’ random slopes for the interaction [log (BF) = 359.1]. We chose this model because the model was the largest one that included all relevant fixed and random structures, and because Barr et al. [39] argued that the maximal random effect structure model is the most precise. Raftery [40] argued that 75–95% posterior probability indicated that the evidence is positive in the Bayesian model selection; therefore, we obtained the mean, density, and 25% lower and upper bound of the posterior samples of the true, false, and void cases (Fig 4). The mean fixed effect parameter and 25% lower bound of the true case was greater than 0, and the mean and 25% upper bound of the false case was smaller than 0. The mean of the void cases was between that of the true and false cases. These results indicated that participants might intuitively interpret that the *TT* was coherent with the conditional statement and may have had positive feelings, that the *TF* was not coherent with the conditional statement and may have had negative feelings, and that the *FT* and *FF* were neither consistent nor inconsistent with the conditional.

## General Discussion

The present study tested whether people intuitively detected the logical value of propositions. We employed an experimental paradigm based on Morsanyi and Handley’s previous studies [18], which asked participants to indicate how they felt when presented with logical propositions and targets. According to the hedonic marker hypothesis, processing fluency evokes positive affect (e.g., Winkielman & Cacioppo [19]) and the semantic coherence of previously presented sentences and target materials, which is called conceptual fluency, evokes positive affect without conscious awareness of the roots of feelings [20]. We hypothesized that a logically true case is semantically coherent with the premise and might be fluently processed and evoke positive affect without conscious awareness. The present results supported this hypothesis. Experiment 1 showed that participants liked true cases more than false cases in four types of propositions (conditional, biconditional, conjunctive, and material implication), and Experiment 2 revealed that the liking ratings of conditionals were affected by not only a matching bias but also by logical principles. Furthermore, the Bayesian mixed meta-analysis regarding the conditional statements revealed that there were differences in the liking ratings for true, false, and void cases. People liked true cases more as compared to false cases, and the liking ratings for void cases fell between those for the true and false cases. These results fit better with the basic notion of logical intuition theories, in that people have implicit knowledge about basic logic and this intuitive logic is automatically activated during reasoning [11,17,41].

Morsanyi and Handley [18] suggested that people intuitively detect the logicality of syllogisms through changes in their affective states. Yet, Klauer and Singmann [21,22] casted doubt on their view. The effect of logical validity on affective states was eliminated when controlling for confounding factors, such as the conclusion content of target items. The present results showed that, even when using abstract letter-number materials, the logical value changed participants’ affective states, thus suggesting that people could intuitively detect logicality.

It is important to note that the reasoning task of Morsanyi and Handley [18] comprised belief-logic manipulated syllogisms that could be more complicated and difficult than the present task. When judging the logical validity of syllogistic reasoning, people need to represent possible cases of premises and test whether the cases fit with the conclusions (e.g., Johnson-Laird [37]), while the present experiment had already presented the target case and participants only checked whether the presented target fit with the premise and logical rules. According to mental model theory, deductive reasoning has the following three steps [42,43]: (1) construction of the initial model from the premise, (2) fleshing out further models and (3) validation of the model by searching for a counter example. The second and third steps of reasoning need cognitive efforts, but the first step is activated automatically during the comprehension process. Therefore, the results of the liking ratings in the present task could be interpreted as being a part of the comprehension process and not that of reasoning. However, the mental model theory of deductive reasoning suggested that initial models of negated statements included affirmative models as well as negated models. For example initial models of negated conditionals, “*if p then not-q”* include both “*[p] not-q*” and “*… q*” models. Therefore, if the liking rating of the present task only reflected the comprehension process then participants would have liked matching cases more than mismatching cases. The results of our meta-analysis indicated that both matching and logical principles affected liking ratings. These results suggest that not only the initial representation of a premise but also its logical coherence affects the intuitive process of reasoning.

Furthermore, the belief bias in syllogistic reasoning is a robust phenomenon, whereby only 56% of participants could make logically correct responses to valid-unbelievable syllogisms [6]. On the other hand, the matching bias in the present experiment was not as strong; 77% of participants gave logically correct answers for true and double mismatching cases. De Neys et al. [41] pointed out that heuristic intuition might be more strongly activated than logical intuition and that complex logical computation might not give rise to logical intuition. Therefore, it is plausible that belief-logic manipulated syllogisms, such as those in Morsanyi and Handley’s studies [18], strongly evoke heuristic intuition and activate logical intuition to a lesser extent, which in turn, diminishes the effect of logicality on affective states. Thompson et al. [16] suggested that easily computable logical deductions, such as Modus Ponens, may give rise to a sense of rightness. In the present experiment, judging the logical value of a basic proposition was easily computed and participants’ implicit logical knowledge might have been more easily activated.

We observed a correlation between explicit reasoning tasks and intuitive judgment: Participants who made more logically correct responses in the truth table task liked logically true cases more, regardless of matching. And the Bayesian mixed meta-analysis indicated there were individual differences in the effect of matching and logicality in the liking rating. These results suggest the presence of individual differences in the strength of logical intuition. Thompson and Johnson [17] revealed that both high- and low-IQ reasoners were sensitive to conflict between logic and heuristics, and their FOR was weaker in conflict problems. They also showed that high-capacity reasoners were more easily involved in the analytic process and made more normative responses for their initial answers. These authors also argued that high-capacity reasoners are more likely to master normative principles and that their logical intuition could be stronger than that of low-capacity reasoners. In the present study, reasoners who made logically correct responses in explicit tasks could have stronger logical intuition, and they liked logically true cases over matching heuristics.

The present study also tested people’s intuitive understanding of the conditional, *if p then q*. The suppositional account of conditional reasoning indicated that interpretation of a natural language indicative conditional is equal to the conditional probability, *q given p*, and that false antecedent cases are void, neither true nor false [24,25,28]. Developmental studies have pointed out that there are three successive interpretation levels for conditionals, namely conjunctive, biconditional and suppositional [44]. Evans et al. [27] argued that conjunctive interpretation of conditionals reflects mental shortcutting or lower cognitive capacity. In the present study, Experiment 1 revealed that the liking rating pattern for conditional statements differed from that of conjunctive, biconditional, or material implication forms, and the results of the meta-analysis indicated that liking ratings for conditionals were best explained by the suppositional account of conditionals. These results suggest that adult reasoners’ intuitive understanding of conditionals is equal to conditional probability, and it is unlikely that reasoners initially understand a conditional as conjunctive, *p and q*, and then engage cognitive effort in order to make suppositional interpretations of the conditional.

Finally, it is important to note that present study could not partial out the influence of deliberate processes in the liking rating task. In present study, the liking rating task required participants to rate their feeling relying on their intuition, and the correlation between the liking rating and rationality score, which relates with participants’ cognitive ability and engaging deliberate thought, was relatively low. However, present experiments did not engage cognitive load and did not use more implicit measures such as response latency and autonomic responses, which were often applied in the intuitive logic studies [45, 46]. Therefore, it is possible that explicit and deliberate reasoning processes affected the present results. For example, the correlation between explicit reasoning performance and liking ratings may reflect individual differences in the intuitive logic, or participants may use their explicit knowledge in the liking rating task. To have a more stringent test for intuitive processes of reasoning, future studies need to conduct tasks under a cognitive load or use implicit measures.

Recent reasoning studies proposed that people possess intuitive logic as well as intuitive heuristics and that these two intuitive processes enable people to intuitively detect heuristic-logic conflicts and engage in analytic processes [11]. The present study indicated that people could intuitively apply their knowledge about the logical value of basic propositions via slight changes in their feelings and that there could be individual differences in the strength of intuitive logic. One must note that the present study asked participants to judge their feelings; therefore, it is possible that participants’ explicit knowledge about logic or ability to monitor their feelings affected the present results. Future studies should use cognitive load tasks or implicit measures, such as dual task, reaction times, or physiological indices to further test intuitive logicality.

## Supporting Information

S1 File Full Results of the Bayesian Mixed Meta-analysis. The excel file contains the full results table of the Bayesian mixed meta-analysis of the liking rating task.
(XLSX)

S2 File Results of the Bayesian Mixed Analysis of Experiment 2. The excel file contains the results table of the Bayesian mixed analysis of the liking rating task in Experiment 2. The overall best model was the same as that used in the meta-analysis: fixed effects of the suppositional * matching and participants random slopes for both main effects of the suppositional and matching.
(XLSX)

S3 File Data of Experiment 1 and 2, and the Meta-analysis. (XLSX)

S4 File The R-script for the Bayesian Mixed Meta-analysis. (R)
